# Computer-Aided Drug Design in Research on Chinese Materia Medica: Methods, Applications, Advantages, and Challenges

**DOI:** 10.3390/pharmaceutics17030315

**Published:** 2025-03-01

**Authors:** Ban Chen, Shuangshuang Liu, Huiyin Xia, Xican Li, Yingqing Zhang

**Affiliations:** 1 Key Laboratory of Fermentation Engineering (Ministry of Education), Cooperative Innovation Centre of Industrial Fermentation (Ministry of Education & Hubei Province), Hubei University of Technology, Wuhan 430068, China; chenban@hbut.edu.cn (B.C.); 102300802@hbut.edu.cn (S.L.); 102410791@hbut.edu.cn (H.X.); 2 School of Chinese Herbal Medicine, Guangzhou University of Chinese Medicine, Guangzhou 510006, China; lixc@gzucm.edu.cn

**Keywords:** computer-aided techniques, computational chemistry, informatics, artificial intelligence, traditional Chinese medicine

## Abstract

Chinese materia medica (CMM) refers to the medicinal substances used in traditional Chinese medicine. In recent years, CMM has become globally prevalent, and scientific research on CMM has increasingly garnered attention. Computer-aided drug design (CADD) has been employed in Western medicine research for many years, contributing significantly to its progress. However, the role of CADD in CMM research has not been systematically reviewed. This review briefly introduces CADD methods in CMM research from the perspectives of computational chemistry (including quantum chemistry, molecular mechanics, and quantum mechanics/molecular mechanics) and informatics (including cheminformatics, bioinformatics, and data mining). Then, it provides an exhaustive discussion of the applications of these CADD methods in CMM research through rich cases. Finally, the review outlines the advantages and challenges of CADD in CMM research. In conclusion, despite the current challenges, CADD still offers unique advantages over traditional experiments. With the development of the CMM industry and computer science, especially driven by artificial intelligence, CADD is poised to play an increasingly pivotal role in advancing CMM research.

## 1. Introduction

Chinese materia medica (CMM) refers to the medicinal substances used in traditional Chinese medicine (TCM) clinical practice and is primarily derived from plants, animals, and minerals. CMM has been used for disease prevention, treatment, and rehabilitation for thousands of years in China. In September 2022, the National Administration of Traditional Chinese Medicine announced that CMM had spread to 196 countries and regions, indicating its growing worldwide acceptance [1]. Nowadays, CMM is recognized by the World Health Organization as a complementary alternative therapy approach [2].

**Figure 1:**
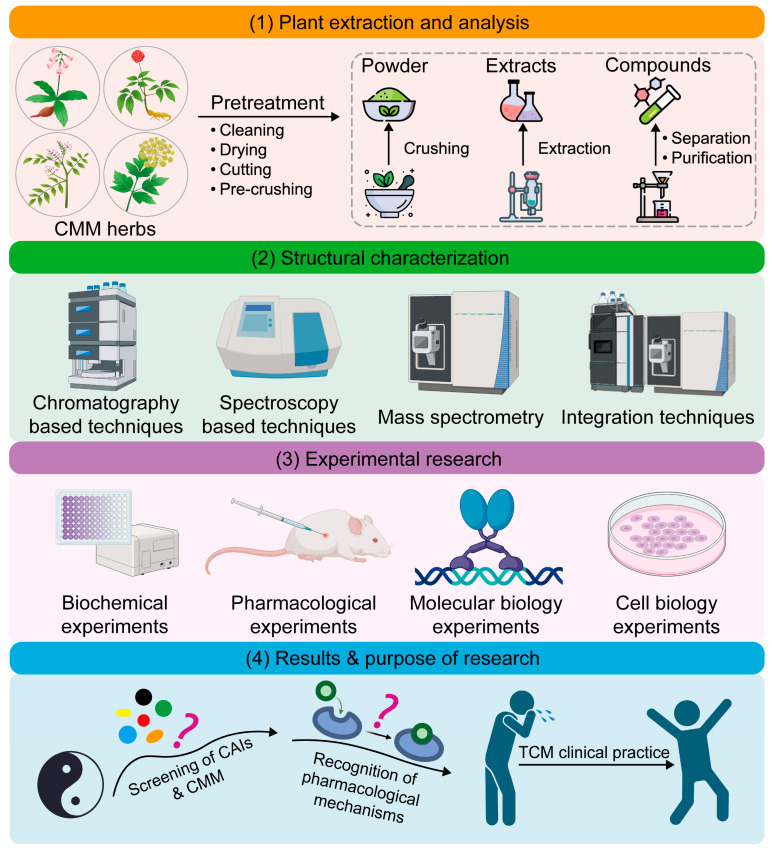
A typical Chinese materia medica (CMM) research workflow and its possible results or purpose. CAIs: CMM active ingredients. TCM: traditional Chinese medicine.

To further facilitate the application and dissemination of CMM, the Chinese government has adopted a supportive stance toward academic research on CMM. Data from the National Natural Science Foundation of China (NSFC) show that the Chinese government has gradually increased its funding for CMM research in recent years. In 2023, the NSFC’s funding for TCM research surpassed CNY 770 million [3]. Moreover, since 2016, the Chinese government has released a series of seminal documents to support the development of TCM [4]. Owing to this official support, academic research on CMM has become a popular scientific field. A typical CMM study may employ analytical techniques to identify specific ingredients from CMM extracts, biochemical experiments to assess the potential activity of these ingredients, and pharmacological experiments to confirm their mechanisms of action (Figure 1) [5]. This approach allows researchers to screen CMM active ingredients (CAIs) and elucidate the properties and actions of a CMM herb.

Nevertheless, CMM formulae are the main means of health maintenance and disease treatment in TCM clinical practice, and they treat diseases through multitarget interventions involving multiple ingredients, differing from Western medical practices. The combination of various CMM herbs in a formula can produce synergistic or antagonistic effects, significantly complicating CMM research [6]. Consequently, typical research methods face significant challenges because of their expense and time-consuming nature, resulting in a relatively weak foundation for CMM research.

To strengthen CMM research, computer-aided drug design (CADD) has been applied in recent years. The concept of CADD was developed as early as the 1970s and it has already been used in Western medicine (WM) research [7]. Initially, CADD referred specifically to molecular simulation techniques based on computational chemistry, which can be subdivided into structure-based drug design (SBDD) and ligand-based drug design (LBDD) [8]. After decades of development, the concept of CADD has expanded significantly, and many studies refer to various computer-aided techniques used in pharmacy research as CADD, including artificial intelligence drug design (AIDD) based on machine learning (ML) and deep learning (DL), which has gained much attention in recent years [9]. Recently, several drugs developed through CADD have been approved by the US Food and Drug Administration (FDA), thereby contributing to the growing prominence of CADD in CMM research [10]. However, due to the relatively late application of CADD in CMM research, there are few comprehensive reviews specifically addressing this topic.

Therefore, this review omits the detailed definition of CADD and instead briefly describes the CMM research-related CADD methods from the two main perspectives of computational chemistry and informatics. For these methods, this review exhaustively elaborates on the application cases that are closely related to CMM research. Finally, the review discusses the advantages and challenges of applying CADD to CMM research. This review will bridge the gap between traditional CMM theories and modern computational approaches, providing a roadmap for future interdisciplinary research.

## 2. Computational Chemistry in CMM Research

Computational chemistry is a branch of theoretical chemistry and a core aspect of CADD. It uses mathematical approximations and computer programs to address specific chemical problems. The computational chemistry methods related to CMM research mainly include quantum chemistry (QC), molecular mechanics (MM), and quantum mechanics/molecular mechanics (QM/MM).

### 2.1. QC

#### 2.1.1. Brief Introduction to QC

QC employs the fundamental principles and methods of quantum mechanics (QM) to study and address chemical problems. QM posits that the motion of microscopic systems can be described by the Schrödinger equation. The solution to this equation, known as the wave function, theoretically describes the motion state of a microscopic system and the various properties determined by this state [11]. Therefore, the development of solving methods for the Schrödinger equation has always been a pivotal aspect in quantum chemical calculation (QCC). Typical QCC methods are primarily divided into semi-empirical methods, ab initio calculations, and density functional theory (DFT). Among these three methods, DFT generally offers a good balance between computational costs and accuracy, establishing itself as the prevailing method in QCC [11].

**Figure 2:**
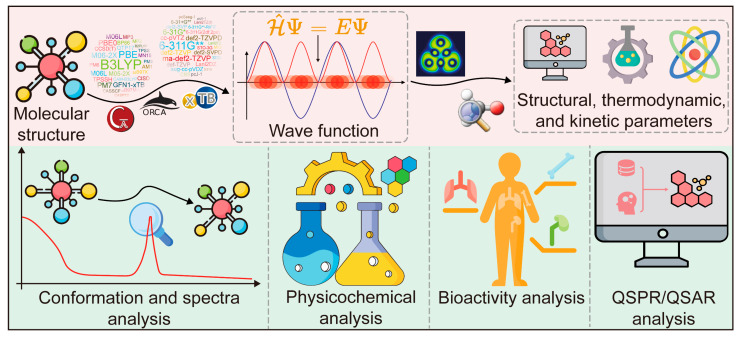
The workflow and application of quantum chemical calculation (QCC) in Chinese materia medica research. QSPR/QSAR: quantitative structure–property relationships and quantitative structure–activity relationships.

The workflow of a QCC study is illustrated in Figure 2. The initial step involves providing the molecular structure, followed by selecting an appropriate computational method. For DFT calculations, the choice of an appropriate functional is critical. After selecting the method, it is usually necessary to set up the base set for the calculation. Some semi-empirical methods, which already have built-in basis sets, do not require additional basis sets. Based on the molecular structure and the chosen method, the QCC program can approximate the wave function, which contains a wealth of chemically significant information, such as structural, thermodynamic, and kinetic parameters. Currently, common QCC programs include Gaussian 09, ORCA 5.0, and xTB 6.7.1 [12,13,14].

#### 2.1.2. Applications of QC in CMM Research

1. Conformation and spectra analysis of CAIs

Investigating the molecular structures of CAIs is crucial in understanding the material basis of CMM. However, the experimental characterization of flexible and complex structures is often challenging. In such cases, conformation searching using QCC offers a robust alternative, facilitating the exploration of potential energy surfaces and the identification of low-energy conformations (Figure 2). Typically, researchers employ less computationally intensive methods, such as MM or semi-empirical methods, for initial structure optimization and energy comparison across numerous conformations. Subsequently, they apply DFT calculations, which are more accurate but computationally demanding, to confirm the dominant conformations [15,16]. Conformational analysis provides precise spatial structural parameters of CAIs, including bond lengths, bond angles, and dihedral angles, often in strong agreement with experimental data. By integrating QCC with experimental data, researchers can achieve enhanced accuracy in characterizing CAIs or complex systems containing them. This significantly reduces the complexity of structural characterization [17]. However, QCC methods based on electronic structure calculations are computationally expensive and unsuitable for large-scale dataset screening. To address these challenges, researchers have developed artificial intelligence (AI) techniques to accelerate the QCC process. Lu et al. introduced Uni-Mol+ (https://github.com/deepmodeling/Uni-Mol/, accessed on 27 February 2025), a DL approach that generates an initial 3D conformation using RDKit (https://www.rdkit.org/, accessed on 27 February 2025) and iteratively refines it toward the DFT-equilibrium conformation via neural networks [18].

Based on accurately characterized conformations, QCC can calculate various spectra of CAIs, including circular dichroism (CD), nuclear magnetic resonance (NMR), Raman, X-ray, ultraviolet–visible (UV–Vis), and infrared (IR) spectra [19,20,21,22,23]. Beyond direct spectral calculations, QCC can also indirectly predict spectra for CAIs. Wang et al. demonstrated a correlation between DFT-calculated energy data and the mass spectrometry (MS)-based fragment abundances of isomeric CAIs, providing a robust theoretical framework for the understanding of the mechanisms underlying fragment ion differences among isomers [24]. Li et al. calculated the dipole moments of isomeric CAIs using DFT, successfully explaining the chromatographic retention times of these isomers [25].

Recent advancements in QCC-based spectral analysis highlight the potential of integrating AI to improve the calculation accuracy while significantly reducing the computational costs [26]. A prominent example is the structure elucidation of natural products (NPs) using ML-assisted quantum chemical NMR calculations, which has emerged as a key area of interest. Tsai et al. applied kernel ridge regression (KRR) to refine NRM shielding constant calculations from DFT, enhancing the precision of the predicted chemical shifts. This approach enables the rapid determination of NP configurations within minutes [27]. Since many NPs also contain CAIs, the incorporation of AI into QCC-based spectral analysis holds significant promise in advancing CMM research.

2. Physicochemical analysis of CAIs

The conformation and spectral analysis of CAIs provide a robust foundation for an understanding of their bioactivity. Nevertheless, structural information alone is insufficient to fully elucidate bioactivity; it is equally important to investigate the physicochemical properties of CAIs.

Over the past decade, researchers have utilized QCC to study the key physicochemical properties of CAIs (Figure 2). Zeng et al. calculated the p*K*a values of 96 carboxylic acids in an aqueous solution, establishing a reliable procedure that accurately reproduces theoretical p*K*a values in close agreement with experimental data [28]. Li et al. employed DFT to calculate the polarity of CAIs, successfully predicting their chromatographic retention times [25]. Guan et al. developed a QC model to predict the octanol–water partition coefficients (Log*P*) of small organic molecules, including CAIs, as part of the SAMPL6 Log*P* Prediction Challenge [29].

Physicochemical properties such as p*K*a, polarity, and Log*P* are relatively straightforward to calculate using QCC. Many other properties require QCC to model specialized systems or parameters for indirect prediction. Yang et al. investigated solute–solvent and solvent–solvent interactions by calculating the solvation free energy and analyzing radial distribution functions to elucidate the solubility of benzoin in three solvent mixtures [30]. Many studies have assessed the stability of CAIs by evaluating the bond energies of key chemical bonds, as well as the energy difference between the highest occupied molecular orbital (HOMO) and the lowest unoccupied molecular orbital (LUMO), which is known as the HOMO–LUMO gap, an approximation of the fundamental gap and optical gap [31,32,33].

The physicochemical properties are closely related to drug-like properties, such as absorption, distribution, metabolism, excretion, and toxicity (ADMET), and are mainly derived from QCC-based structural and thermodynamic parameters [34]. Additionally, QCC-based kinetic parameters can provide valuable insights into the physicochemical properties of CAIs. For instance, calculations of the transition states, intrinsic reaction coordinates (IRC), and reaction rates provide a deeper understanding of the reactivity of CAIs. Such insights elucidate their biosynthetic pathways in vivo and inform their synthesis and structural modification in vitro [32,35,36].

In fact, based on structural, thermodynamic, and kinetic parameters, QCC can predict an even broader range of physicochemical properties, such as the redox potential, density, enthalpy of evaporation, boiling point, polarizability, and magnetic moment. These predictions are widely applied in WM research and related disciplines [37,38,39,40,41,42,43]. Since both CAIs and WMs are fundamentally chemical molecules, QCC is poised to play an increasingly significant role in the physicochemical analysis of CAIs.

3. Bioactivity analysis of CAIs

Traditional physicochemical properties are typically measurable through experimental methods. However, the wave functions generated by QCC can be analyzed to derive numerous QC parameters that, while purely theoretical, are essential in evaluating the bioactivity of CAIs (Figure 2). Wave function analysis is commonly performed using software tools such as GaussView 6 and Multiwfn 3.8 [44,45].

Chen et al. confirmed the antioxidant activity, sites, mechanisms, and products of several CAIs via experiments and various QC parameters, such as molecular surface electrostatic potentials (ESP), electron density differences, and spin densities [32]. Liu et al. investigated the natural population analysis (NPA) charges, frontier molecular orbitals, molecular ESP, and chemical reactivity descriptors for evodiamine and rutaecarpine, based on which the potential anti-cancer activity and mechanisms of the CAIs were predicted [46]. Zhang et al. predicted the anti-inflammatory activity of several CAIs by elucidating their interactions with related targets based on the ESP, HOMO energy, and LUMO energy [47]. QC parameters also facilitate the evaluation of a broad spectrum of bioactivity, including anti-viral, anti-obesity, and anti-diabetic effects. These evaluations are primarily conducted by analyzing the physicochemical properties of CAIs and their interactions with biological targets [48,49,50].

4. Quantitative structure–property relationship (QSPR) and quantitative structure–activity relationship (QSAR) analysis of CAIs

Since the physicochemical properties and bioactivity of CAIs can be analyzed using QCC, many studies have tried to predict the physicochemical properties and bioactivity of molecules using QCC-aided mathematical models. These models are referred to as quantitative structure–property relationship (QSPR) and quantitative structure–activity relationship (QSAR) models, respectively (Figure 2).

In QCC-aided QSPR/QSAR studies, the structural, thermodynamic, kinetic, and QC parameters are commonly used as descriptors. These descriptors serve as input variables in statistical models to establish correlations with specific properties or types of activity, enabling the creation of predictive models that inform drug design and development. Currently, QCC-based QSPR/QSAR studies focused on CAIs remain limited, with only a handful published sporadically over the past two decades. This scarcity may be attributed to the time-intensive nature of QCC and experimental research on large datasets of CAIs [51,52,53,54].

Conversely, QCC-aided QSPR/QSAR studies are significantly more prevalent in WM and other disciplines, such as analytical, materials, and environmental chemistry. These fields benefit from the compatibility, clear physicochemical relevance, and high accuracy of QCC descriptors [53]. Notable advancements have been made, particularly in the development of databases and online tools that incorporate QC descriptors into predictive modeling processes [55,56,57]. The integration of ML and DL algorithms into QSPR/QSAR analysis has further enhanced the predictive accuracy and applicability by capturing complex nonlinear relationships and managing large-scale, high-dimensional data [58]. With the availability of such databases and tools, researchers often favor direct property or activity predictions over de novo QSPR/QSAR modeling for CAIs [31,59].

### 2.2. MM

#### 2.2.1. Brief Introduction to MM

QCC is inherently complex and primarily applied to small molecules. MM relies on classical Newtonian mechanics, using simplified functional forms to describe potential interactions between atoms [60]. Unlike QCC, MM does not account for electronic motion, treating entire atoms—or even groups of atoms—as single particles. The simplicity of MM enables extremely rapid calculations, making it particularly suitable for studying large molecular systems.

The core of MM lies in the development of a force field, which consists of energy terms and their specific functional forms. These energy terms are typically categorized into bonding and non-bonding terms. Bonding terms represent interactions between adjacent atoms within a molecule, while non-bonding terms capture interactions between molecules and between non-adjacent atoms within a molecule. Several molecular force fields commonly used in CMM-related molecular systems are summarized in Table 1. When MM calculations incorporate the temporal evolution of molecular states, the method is referred to as molecular dynamics (MD). MD solves Newton’s equations of motion to determine the positions and velocities of atoms over time, providing dynamic insights into molecular behavior [61]. Research methodologies derived from MM and MD, such as molecular docking and molecular dynamics simulation (MDS), are collectively known as molecular simulation techniques.

Molecular docking is a technique used to predict interactions between two or more molecules. Widely used molecular docking programs include AutoDock 4.2.6, AutoDock Vina 1.1.2, Schrödinger Glide 2024-4, MOE 2019, and DOCK 4.0 [91,92,93,94,95]. The typical process for the use of these programs involves the following steps: (1) the preparation and preprocessing of the ligand structure (e.g., a medicinal small molecule) and the receptor structure (e.g., a biological macromolecule); (2) setting the docking parameters, such as searching algorithms, the number of runs, and docking areas; (3) searching for the optimal binding modes and calculating the docking score, a numerical value that quantifies the binding affinity; (4) analyzing and visualizing the results, including the system structure, binding sites, and binding mechanisms (e.g., hydrogen bonding, hydrophobic interactions, and electrostatic interactions).

**Figure 3:**
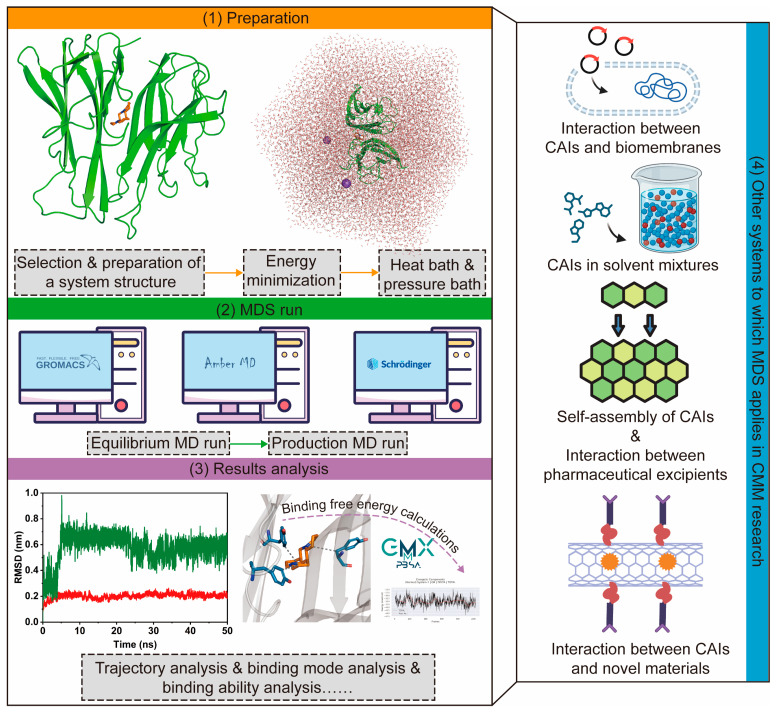
The molecular dynamics simulation (MDS) process (**1**–**3**) for a typical ligand–receptor complex. Other systems to which MDS is applied in Chinese materia medica (CMM) research are also shown in (**4**). CAIs: CMM active ingredients.

In subsequent studies involving molecular docking, MDS is commonly employed. This is because molecular docking generates a static model of the ligand–receptor complex, which cannot capture the dynamic nature of the binding process in a specific environment. MDS addresses the limitations of molecular docking and traditional experiments by offering insights into the dynamic behavior of molecular interactions over time, under specified conditions regarding the solvent, temperature, and pressure. MDS is typically performed using specialized programs like Amber 24, Gromacs 2020.3, and GROMOS 1.6.0, with the basic workflow depicted in Figure 3 [96,97,98].

#### 2.2.2. Applications of MM in CMM Research

1. Virtual screening of CAIs

Over the past two decades, more than 100,000 publications on molecular docking have been recorded across platforms such as Google Scholar, Web of Science, PubMed, and the China National Knowledge Infrastructure (CNKI). Notably, approximately half of the 20,000 CNKI publications originate from the field of traditional Chinese pharmacy, highlighting the extensive application of molecular docking in CMM research.

Since its introduction in the 1970s, molecular docking has become a cornerstone technique in CADD. In SBDD, virtual screening plays a pivotal role, leveraging molecular docking and other computational tools to identify potential drug candidates. When target information is available, molecular docking facilitates the identification process. Conversely, when target information is unavailable but drug activity is known, MM-based molecular descriptors can be utilized for QSPR/QSAR analysis, which constitutes a primary approach in LBDD. Recently, the widespread adoption of MM-derived methods has enabled the integration of both SBDD and LBDD into CMM research [99,100]. While the key steps of QSPR/QSAR analysis in LBDD were discussed in the preceding section, this section focuses on virtual screening within the framework of SBDD.

Literature research indicates that molecular docking has been employed for the virtual screening of CAIs since the early 2000s. In 2004, Zhang et al. elucidated the pharmacological mechanism of a natural potassium channel blocker derived from the venom of *Buthus martensii* Karsch [101]. Two years later, in 2006, Gao et al. used human immunodeficiency virus (HIV)-1 protease as a receptor to screen potential anti-HIV drugs from a TCM database [102]. Despite the time that has elapsed since these studies, they continue to exemplify two primary applications of molecular docking in CMM research: (1) the virtual screening of CAIs based on binding mechanisms and (2) the virtual screening of CAIs based on binding affinities. The former approach relies on the structural characteristics of docked complexes, while the latter utilizes quantitative metrics, such as scoring functions, to evaluate the binding ability.

In recent years, molecular docking has undergone significant advancements, primarily in four key areas: enhanced computational efficiency, improved scoring functions, expanded docking systems (e.g., flexible macromolecules, metal-containing molecules, and covalent-linked complexes), and increased interdisciplinary integration (e.g., combining molecular docking with QC, AI, and big data) [103,104]. These developments have also contributed to the progress of virtual screening for CAIs [105,106].

As previously noted, MDS is frequently employed as a follow-up to molecular docking studies. Using trajectories generated from MDS, the binding free energy of a ligand–receptor complex can be calculated and decomposed through methods such as thermodynamic integration, free energy perturbation, linear interaction energy, and molecular mechanics Poisson–Boltzmann/generalized Born surface area (MM/PB(GB)SA, Figure 3) [107,108,109,110]. From a theoretical perspective, MDS and binding free energy calculations offer greater accuracy compared to molecular docking but are significantly more complex and computationally intensive. As a result, researchers commonly adopt a tiered approach: molecular docking is used for initial high-throughput screening, followed by MDS and experimental validation to refine the screening process. This integrated strategy has been extensively applied in the virtual screening of CAIs [111,112].

2. MDS for various systems related to CMM research

The virtual screening of CAIs typically requires known targets, such as proteins, enzymes, and DNA. However, the interactions between CAIs and their targets are not always limited to virtual screening purposes. Zhang et al. utilized MDS to analyze the intermolecular interactions between various CAIs and bovine serum albumin, providing insights into CAIs’ penetration during the cross-flow ultrafiltration of CMM solutions [113]. In fact, when studying the penetration effects of CAIs, more studies select bio-membranes rather than proteins as models (Figure 3). These studies have not only enhanced the bioavailability of CAIs but also facilitated the development of penetration enhancers derived from CAIs [114,115,116]. In addition to penetration enhancers, other CMM-related pharmaceutical excipients have been explored using MDS. Zhou et al. employed MDS to simulate the interaction mechanism between binders and powders, offering guidance for the industrial production of CMM granules [117]. Shen et al. used MDS to screen the excipients and investigate the blend system of ginsenoside K nanostructured lipid carriers [118]. Dai et al. used MDS to study the self-assembly process of ginsenoside Ro, providing a theoretical foundation for vesicle formation in ginsenoside Ro and other CMM saponins, which show potential as surfactants and solubilizers [119]. These studies highlight the applicability of MDS for CAIs in complex solution systems such as solvent mixtures, CAIs interacting with pharmaceutical excipients, and the self-assembly of CAIs (Figure 3).

The self-assembly of CAIs occurs frequently, both in vitro and in vivo. Lei et al. performed MDS to simulate the self-assembly of berberine and glycyrrhizic acid, revealing that the process is driven by hydrogen bonding, π–π stacking, and electrostatic interactions to form a hydrogel. This hydrogel demonstrated injectability, safety, favorable release properties, and a significant anti-inflammatory effect in treating ulcerative colitis [120]. Zhang et al. provided evidence that supramolecular pigments could form in vivo during CMM metabolism via simultaneous covalent and non-covalent assembly, potentially playing crucial roles in pharmacological activity. Their study employed coarse-grained and all-atom MDS, both of which aligned well with the experimental findings [121]. CAIs can self-assemble not only with other CAIs but also with molecules such as organic acids and metal ions. These interactions, elucidated through MDS, result in advanced bioactive materials like nanoparticles, micelles, and gels [122]. Derived from renewable resources, these materials are easy to prepare and multifunctional, offering properties like controlled release, smart-responsive release, and potent biological effects for the treatment of various diseases [122]. Thus, such materials retain the benefits of CAIs while overcoming their limitations.

Despite its growing application in CMM research, MDS faces several limitations, including insufficient sampling, inaccuracies in atomistic models, and challenges in analyzing and interpreting trajectories [123]. Recent advances in AI, particularly in ML and DL, present opportunities to address these challenges. Developments in AI have led to the creation of AI-based force fields, improved techniques for conformational space sampling, and innovative methods for trajectory analysis [123].

### 2.3. QM/MM

#### 2.3.1. Brief Introduction to QM/MM

QM is well-suited for the calculation of properties related to electron motion, such as electronic structures, spectroscopic properties, molecular orbitals, and chemical bond breaking. In contrast, MM is more appropriate for the analysis of the conformations and motions of large molecules. Due to their differing computational principles, QC offers higher accuracy but at the expense of slower computation, whereas MM calculations are faster but less precise.

**Figure 4:**
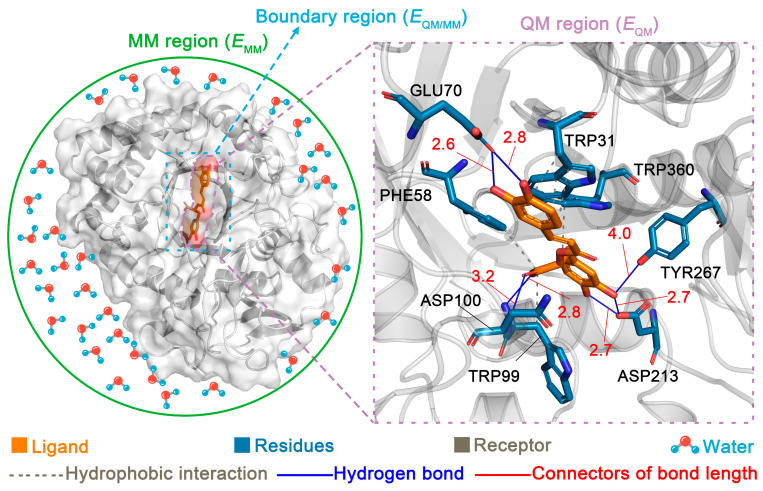
Representation of the quantum mechanics/molecular mechanics method for a ligand–receptor system.

To combine the strengths of QM and MM, Warshel and Levitt introduced the QM/MM method in 1976 [124]. This hybrid approach partitions the system into three regions: the QM region, the MM region, and the boundary region (Figure 4). The total energy of the system is defined as the sum of the energies of these three components. QM methods are applied to regions where chemical changes occur, such as enzyme binding sites or substrate interactions (i.e., the QM region). MM methods handle the remainder of the system, including receptors and solvent molecules (i.e., the MM region). To address the interface between QM and MM regions (i.e., the boundary region), techniques such as atom-linking methods, boundary atom methods, or local orbital methods are employed to ensure accurate and seamless integration [124]. Currently, there are many QCC and MDS programs that can perform QM/MM calculations independently, such as Gaussian 09, ORCA 5.0, CP2K 2025.1, and Amber 24 [13,96,125]. To combine the strengths of different programs, researchers prefer to use QCC programs in conjunction with MDS programs to perform QM/MM calculations, such as by coupling Gaussian 09 and Gromacs 2020.3 [126]. To facilitate this combination, researchers have also worked on the development of specialized packages to call QCC and MDS programs and integrate commonly used options in QM/MM calculations [127].

#### 2.3.2. Applications of QM/MM in CMM Research

In CMM research, QM/MM is frequently used to simulate the interactions between CAIs and proteins. Due to the application of QCC for key regions, QM/MM offers higher accuracy than MDS alone, providing a deeper understanding of the binding modes between CAIs and the target proteins [128]. A novel molecular docking method based on the QM/MM theory, known as QM-polarized ligand docking, is more accurate than traditional molecular docking. This method is useful for the virtual screening and QSPR/QSAR analysis of CAIs [129]. Moreover, QM/MM is particularly well suited for studying interactions between CAIs and enzymes, offering a powerful tool for the investigation of enzymatic reactions in CMM systems [130,131]. This capability is especially valuable in synthetic biology, where enzymatic reactions in CMM systems guide the biosynthesis pathways of target CAIs [126,132].

## 3. Informatics

Computational chemistry and traditional experimental approaches have generated vast amounts of data in CMM research. Efficient data processing methods have, in turn, significantly advanced the field of computational chemistry. Cheminformatics and bioinformatics now provide robust theoretical frameworks and practical tools for data processing, while data mining has further strengthened the connection between raw data and actionable information in practical applications. Collectively, these disciplines form the informatics foundation of CADD.

### 3.1. Cheminformatics and Bioinformatics

#### 3.1.1. Brief Introduction to Cheminformatics and Bioinformatics

Cheminformatics leverages computational tools to represent, manage, analyze, simulate, and disseminate chemical information. One of the primary challenges in this field is efficiently parsing large datasets to extract meaningful molecular information, making the accurate representation of molecular structures a critical focus. This representation is also a prerequisite for computational chemistry studies of CAIs. As demonstrated in the aforementioned cases, cheminformatics was integral to the studies conducted by Zhang and Gao, who utilized 3D conformations and molecular fingerprints, respectively, to represent CAIs. Their computational and experimental studies, grounded in these representations, ultimately achieved their intended objectives [101,102]. Such approaches have become commonplace in recent decades, converting molecular structures into various machine-readable formats, including the simplified molecular input line entry system (SMILES), International Chemical Identifier (InChI), molecular fingerprints, and graphical representations. These formats facilitate the analysis and comparison of molecular structures, as well as virtual screening and QSPR/QSAR analyses for CAIs [133,134].

**Figure 5:**
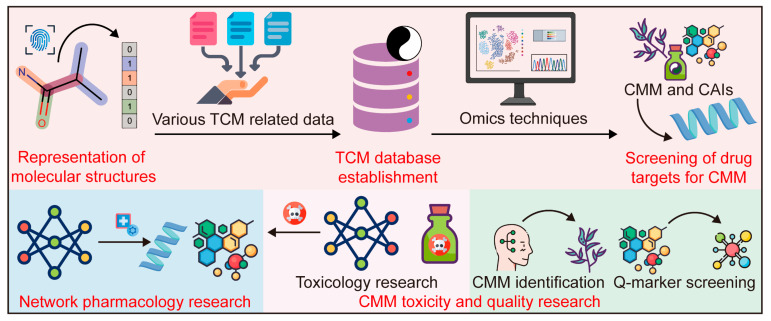
Applications of cheminformatics and bioinformatics in Chinese materia medica (CMM) research. TCM: traditional Chinese medicine; CAIs: CMM active ingredients; Q-marker: quality marker.

Initially developed to streamline the drug discovery and development process, cheminformatics has since grown to play an increasingly vital role in biology and biochemistry [135]. From this point of view, cheminformatics and bioinformatics are almost inseparable as the cheminformatics approach is a necessary pathway for bioinformatics, which involves the use of computational methods to analyze biological data. Given this inherent overlap, in addition to the representation of molecular structures by means of cheminformatics, the subsequent application cases will no longer strictly distinguish between cheminformatics and bioinformatics (Figure 5).

#### 3.1.2. Application of Cheminformatics and Bioinformatics in CMM Research

1. TCM database establishment

In CMM research, the integration of cheminformatics and bioinformatics has revolutionized how chemical and biological data are explored and utilized, particularly within the realm of TCM databases [136]. Today, most TCM databases not only include chemical information about CAIs but also encompass TCM-related biological data. Furthermore, some TCM databases provide additional information on botanical sources, formulae, clinical cases, and TCM theories. These comprehensive databases have been made possible by the advanced data processing capabilities of cheminformatics and bioinformatics.

For a long time, TCM databases have significantly contributed to CMM research by offering a valuable source of TCM-related information. Unfortunately, many of these databases have either ceased to be maintained or are no longer accessible. We have compiled a list of free TCM databases and tested their accessibility (Table 2). It is important to note that all the databases in Table 2 explicitly focus on TCM-related information, rather than covering a broader range of NPs. Additionally, we have only included comprehensive TCM databases and excluded others focused on specific diseases, as the number of such databases is too large to cover in this paper. Other well-known databases based on cheminformatics and bioinformatics, such as RCSB PDB (https://www.rcsb.org/, accessed on 27 February 2025), NCBI (https://www.ncbi.nlm.nih.gov/, accessed on 27 February 2025), and GeneCards (https://www.genecards.org/, accessed on 27 February 2025), are not included in our list because they are not specialized TCM databases [136].

While TCM databases provide invaluable resources for CMM research, the discontinuation or inaccessibility of many highlights the urgent need for the sustainable management and continuous updating of these repositories. Moreover, recent trends show growing homogeneity in the development and application of these databases. Several studies have indicated significant data overlap between databases, with some even containing notable errors when compared to experimental results [137]. There is a pressing need to ensure clear and reliable data sources when incorporating them into databases.

**Table 2:** Specialized databases for traditional Chinese medicine research.

Database	Number of Formulae	Number of Herbs	Number of Ingredients	Number of Targets	Number of Cases	Website	Accessibility
BATMAN-TCM [138]	54,832	8404	39,171	2,319,272	-	http://bionet.ncpsb.org.cn/batman-tcm/#/home	Yes
CancerHSP [139]	-	2439	3575	-	-	https://old.tcmsp-e.com/CancerHSP.php	Yes
CEMTDD [140]	-	621	4060	2163	-	http://www.cemtdd.com/index.html	No
CMAUP [141]	-	7865	60,222	758	-	https://www.bidd.group/CMAUP/	Yes
CMCR	-	-	-	-	111,653	https://cmcr.yiigle.com/	Yes
CPMCP [142]	656	1560	27,928	20,965	-	http://cpmcp.top	No
ETCM [143]	3959	402	7284	2266	-	http://www.tcmip.cn/ETCM/	Yes
ETM-DB [144]	573	1054	4285	-	-	http://biosoft.kaist.ac.kr/etm/home.php	No
Herb [145]	-	7263	49,258	12,933	-	http://herb.ac.cn/	Yes
HIT [146]	-	1250	1237	2208	-	http://www.badd-cao.net:2345/	Yes
IGTCM [147]	-	83	1033	-	-	http://yeyn.group:96/	Yes
iTCM [148]	25,875	8454	43,430	18,851	-	http://itcm.biotcm.net/	Yes
LTM-TCM [149]	48,126	9122	34,967	13,109	-	http://cloud.tasly.com/#/tcm/home	No
SuperTCM [150]	-	6516	55,772	543	-	http://tcm.charite.de/supertcm	Yes
SymMap [151]	-	698	25,975	20,965	-	https://www.symmap.org	No
TCM@taiwan [152]	-	352	37,170	-	-	http://tcm.cmu.edu.tw/	No
TCMBank [153]	-	9193	61,965	32,529	-	https://tcmbank.cn/	No
TCM-ID [154]	7443	2751	7375	768	-	https://www.bidd.group/TCMID/	Yes
TCMID [155]	99,582	10,846	43,413	-	-	https://www.megabionet.org/tcmid/	No
TCMIO [156]	1493	618	16,437	400	-	http://tcmio.xielab.net	Yes
TCMIP V2.0	3959	402	7284	2266	-	http://www.tcmip.cn/	Yes
TCMM [157]	48,043	8932	69,816	76,449		www.tcmm.net.cn/zh-hans/	Yes
TCM-Mesh [158]	-	6235	383,840	-	-	http://mesh.tcm.microbioinformatics.org/	No
TCMSID [159]	-	499	20,015	3270	-	https://tcm.scbdd.com/	No
TCMSP [160]	-	499	29,384	3311	-	https://old.tcmsp-e.com/tcmsp.php	Yes
TCMSSD [161]	133,518	8259	43,413	17,602	-	http://tcmssd.ratcm.cn/	Yes
TCM-suite [162]	6692	7322	704,321	-	-	http://tcm-suite.aimicrobiome.cn/	Yes
TM-MC [163]	5075	635	34,656	13,971	-	https://tm-mc.kr/material.do	Yes
YaTCM [164]	1813	6220	47,696	18,697	-	http://cadd.pharmacy.nankai.edu.cn/yatcm/home	No
Imedbooks	95,260	8980	-	-	-	https://www.imedbooks.com/	Yes
TCMkb	-	-	-	-	465,784	http://www.tcmkb.cn/consiliaweb/	Yes
Shoudao Zhongyi	-	-	-	-	400,000	https://www.shoudaozhongyi.com/	Yes
Yian	586	895	-	-	30	http://www.zhongyoo.com/yian/	Yes
Yideng Xuyan	400,000	-	-	-	102,000	http://db.yidxy.com/prescriptions	Yes
TCMdoc	80,000	11,239	-	-	60,000	http://www.tcmdoc.cn/YiAn/index.aspx	Yes

Note: Many databases specializing in Chinese medicine are not listed in the table because they are accessible for a fee or the statistics are unavailable. The accessibility of all websites was tested on 27 February 2025. The “-” means no data.

2. Screening of drug targets for CMM

Due to the vastness of TCM databases and beyond, researchers have developed a range of bioinformatics-based methods to screen drug targets for CMM. These methods often integrate computational chemistry with omics techniques, particularly genomics, transcriptomics, and proteomics, to systematically analyze the molecular characteristics and network relationships within the genome, transcriptome, and proteome.

Genomics provides a comprehensive approach to annotating and analyzing genomic data, which facilitates the identification of potential targets for CAIs. Gene ontology (GO) and pathway enrichment analysis are commonly used to link genes with specific biological functions and pathways relevant to CMM [165]. Moreover, genome-wide association studies (GWAS) and network-based methods help to uncover gene–disease associations, establishing a systematic framework for the identification of CMM-targeted genes [166]. Transcriptomics focuses on the study of RNA transcripts, enabling the investigation of gene expression changes induced by CMM treatments [167]. High-throughput RNA sequencing has become an essential tool in transcriptomics, allowing for the identification of differentially expressed genes (DEGs) associated with specific CAIs [168]. Network-based methods like weighted gene co-expression network analysis (WGCNA) provide insights into regulatory modules and their potential drug–target relationships [169]. Proteomics examines the complete set of proteins expressed in a biological system and their dynamic interactions. As CAIs often interact directly with proteins, proteomics plays a crucial role in target prediction. Computational chemistry supports the virtual screening of CAIs against potential protein targets, helping to identify high-affinity interactions [170]. Protein–protein interaction (PPI) networks, constructed using bioinformatics platforms like Cytoscape 3.10.3, allow for the visualization and analysis of CMM-induced changes in protein networks [171,172].

Researchers increasingly prefer to use multi-omics techniques to screen targets for CAIs, rather than relying on specific omics methods. For instance, Guo et al. used a DL model based on graph neural networks to predict and discover novel berberine derivatives that could target *Helicobacter pylori*. The efficacy and mechanisms of the predicted CAIs were verified using pharmacokinetic and multi-omics approaches [173]. In addition to screening potential targets for individual CAIs, multi-omics techniques are often combined with AI to identify targets for CMM herbs, CMM formulae, and Chinese patent medicines [174,175,176].

3. CMM network pharmacology (CMM-NP) research

Screening marker drug targets is useful in identifying CAIs and assessing their mechanisms of action. However, this approach alone is insufficient to fully characterize the efficacy and mechanisms of CMM, which exerts its therapeutic effects through multiple CAIs, multiple targets, and multiple pathways. Given the complexity of the signaling networks involved in CMM’s treatment of diseases, a network-based approach is essential for CMM research. In 2007, Li et al. utilized bioinformatics to construct the first biomolecular network for Cold/Hot syndrome in TCM, revealing the network’s regulatory effects of formulae for Cold/Hot syndrome [177]. In the same year, “network pharmacology” was introduced by Andrew L. Hopkins, a pharmacologist at Dundee University in the UK [178].

In the following decades, the application of network pharmacology in CMM research grew rapidly, leading to the emergence of a specialized field known as CMM-NP. A search using the terms “network pharmacology” and “Chinese medicine” in Web of Science reveals over 4700 publications. In the CNKI, searching for “network pharmacology” yields over 18,600 publications in Chinese, more than 16,000 of which are from the field of traditional Chinese pharmacy. These statistics demonstrate that network pharmacology, as a bioinformatics-based research method, has become a pivotal tool in CMM research since its inception.

CMM-NP research generally follows several steps: (1) the identification of CAIs and their potential targets through literature mining and database screening (e.g., the TCM databases listed in Table 2 and other public databases); (2) the use of bioinformatics tools and databases such as GeneCards and OMIM (http://www.omim.org/, accessed on 27 February 2025) to obtain disease targets and construct disease–CAI–target networks; (3) the construction of PPI networks using target data from databases like STRING (https://cn.string-db.org/, accessed on 27 February 2025) to explore potential molecular mechanisms; functional annotation, including GO and Kyoto Encyclopedia of Genes and Genomes (KEGG) pathway enrichment analysis, is then conducted to investigate the biological significance of key targets and related pathways; (4) molecular simulations are employed to validate the interactions between key targets and CAIs, providing theoretical support for an understanding of the mechanisms of CMM.

Compared to the single-omics techniques used to screen drug targets for CAIs, CMM-NP offers a significant throughput advantage, as it can screen multiple CAIs, targets, and pathways simultaneously. These CAIs, targets, and pathways form a complex network, facilitating the systematic exploration of the pharmacodynamics, material basis, and mechanisms of action of CMM [179,180]. Peng et al. applied CMM-NP and experimental investigations to explore the material basis and potential molecular mechanisms of a CMM pair containing *Astragali radix* and *Spatholobi caulis* in the treatment of bone marrow suppression. They identified 36 CAIs from the CMM pair and screened eight potential drug targets, suggesting that the CMM pair may be used to treat myelosuppression through various biological processes [181]. Similarly, CMM-NP has been applied in the study of CMM formulae and Chinese patent medicines. In these cases, different CMM herbs are considered as additional nodes in the “disease–CMM herb–CAI–target–pathway” network, providing a novel approach to uncovering and visualizing the underlying interaction networks of CMM in the treatment of multifactorial diseases [182,183].

The holistic and systematic nature of CMM-NP aligns with the overall concept and dialectical treatment of CMM, making it widely applicable for the screening of CAIs, the prediction of targets, and the interpretation of the mechanisms of action of CMM. This approach holds great promise in uncovering new insights in the field of CMM research. With the advent of advanced techniques, particularly omics and AI, CMM-NP has been refined and deeply implemented, demonstrating its potential as the next paradigm in drug discovery [179,180].

4. CMM toxicity and quality research

Researchers have developed a technique akin to CMM-NP, known as network toxicology, which focuses on toxic CAIs and their associated targets as nodes in the network [184]. This approach typically integrates omics, molecular simulations, and AI to predict the toxicity of CMM and explain the underlying mechanisms through network topology analysis. In the past five years, most network toxicology research in CMM has centered on predicting cardiotoxicity, hepatotoxicity, neurotoxicity, reproductive toxicity, nephrotoxicity, and brain toxicity [185,186,187,188,189,190]. Beyond network toxicology, several bioinformatics-based methods have been developed to predict CMM toxicity, falling into three main categories: (1) traditional QSPR/QSAR models based on computational chemical descriptors, (2) AI models based on available toxicity data from databases and the literature, and (3) AI models based on traditional CMM toxicity theories documented in ancient texts [191,192,193].

Toxicity is a key indicator in evaluating CMM’s safety, while another critical indicator is the CMM quality. Currently, adulteration is widespread in the CMM industry, making the rapid and accurate identification of CMM essential for quality control [194]. Fortunately, CADD technologies, particularly those based on bioinformatics and AI, have enabled significant progress in CMM quality studies. Genomic, transcriptomic, and proteomic data for many CMM herbs are recorded in the GenBank database, greatly assisting in the identification of CMM herbs. For example, DNA barcoding, a bioinformatics-based technology, has gained significant attention in recent years and is widely used for the identification and characterization of CMM herbs. It has also been included in the *Chinese Pharmacopoeia* [195]. In the CMM industry, CMM herbs with excellent quality, produced in a specific region, are usually referred to as geo-authentic (*Daodi* in Chinese) herbs. For a long time, the formation mechanisms of geo-authentic herbs have posed challenges for traditional experimental research, but bioinformatics is expected to reduce these difficulties. By analyzing the omics data of CMM herbs, bioinformatics can help to reveal the genetic background and germplasm resource differences of geo-authentic herbs, explore the influence of the environment on gene expression, and identify metabolic markers that are characteristic of production areas [196,197].

The analysis of metabolic markers, often serving as CAIs, is crucial in ensuring CMM quality. In 2016, academician Chang-xiao Liu introduced the concept of quality markers (Q-markers), which are compounds that serve as comprehensive quality indicators [198]. Unlike ordinary CAIs, Q-markers should not only represent the pharmacological activity of CMM but also offer additional characteristics, such as testability, specificity, traceability, and TCM relevance [31]. Since the introduction of the Q-marker concept, research in this area has grown rapidly, contributing positively to the quality evaluation and control of CMM. Through a systematic literature review, we found that CADD techniques, particularly bioinformatics methods, are frequently employed in Q-marker research for CMM. These methods primarily screen key CAIs through CMM-NP and omics, combined with traditional experiments to evaluate whether the CAIs meet the principles of Q-markers [31]. Some studies have even introduced AI algorithms for the automated prediction of Q-markers, although these efforts are still in the preliminary stage [199].

### 3.2. Data Mining

#### 3.2.1. Brief Introduction to Data Mining

**Figure 6:**
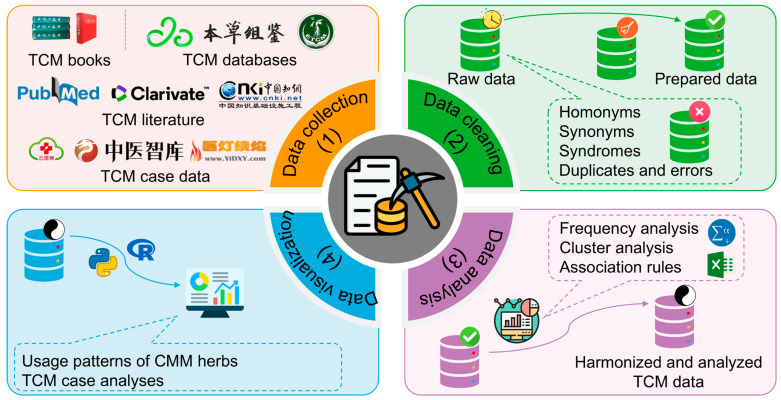
The data mining workflow in Chinese materia medica (CMM) research. TCM: traditional Chinese medicine.

Data mining is the process of extracting meaningful information and patterns from large datasets, making it a crucial component in the fields of data science and computer science. It integrates techniques from statistics, AI, and database management to uncover potential knowledge, relationships, and trends. In the context of CADD, data mining plays a vital role in conjunction with cheminformatics and bioinformatics. This is particularly relevant due to the complexity and vastness of chemical and biological data related to drugs, requiring sophisticated scientific data processing techniques to extract valuable insights. Data mining provides the necessary methodology for this process, with key steps including data collection, data cleaning, data analysis, and data visualization (Figure 6).

#### 3.2.2. Application of Data Mining in CMM Research

In fact, data mining is integral to many of the applications discussed in the previous section. Its use in conjunction with computational chemistry, cheminformatics, and bioinformatics for CMM research is widespread. For instance, data mining plays a crucial role in the establishment of TCM databases, CMM-NP research, multi-omics studies of CMM, and toxicology studies of CMM. In this section, we focus on other significant aspects of data mining in CMM research.

Analyzing the usage patterns of CMM is a common task in data mining for CMM research. By examining these patterns, researchers can gain insights into the interactions between different CMM herbs, optimizing CMM formulae, improving their therapeutic effects, and guiding clinical practice [200,201,202]. The primary raw data sources include the literature, case reports, books, and TCM databases (Figure 6 and Table 2). However, raw data often contain noise due to various factors: (1) homonyms (i.e., different CMM herbs with the same name) and synonyms (i.e., the same CMM herb with different names) are common in TCM clinical practice; (2) diseases in modern medicine may have various names in traditional medicine, referred to as “syndromes” (*Zhenghou* in Chinese); and (3) raw data may include duplicates and errors. To ensure the usability of the data, multiple rounds of data cleaning by different individuals are essential.

Data analysis involves statistical techniques, such as frequency analysis, cluster analysis, and association rule mining (Figure 6). This process often utilizes specialized software, including common tools like IBM SPSS Statistics 26 and Excel 2016, as well as dedicated platforms for the analysis of CMM formulae data, such as the TCM Inheritance Support System Software Platform V2.5, the Ancient and Modern Medical Cases Cloud Platform V2.3.5, TCM Miner (https://www.tcmminer.com/, accessed on 27 February 2025), and ITCMDAS (https://gitee.com/serendipity_LB/itcmdas, accessed on 27 February 2025) [203,204,205,206]. Based on the results, researchers typically use software or programming languages (e.g., Python 3.10 and R 4.4.3) to visualize the findings through charts (Figure 6).

Data mining is also frequently applied in large-scale case analyses of TCM clinical practice, which significantly enhances medical quality, supports medical education, and informs clinical practice (Figure 6). Guo et al. used data mining to study a ten-year dataset and found that younger people with higher education levels in China are more likely to use CMM when ill [207]. Similarly, Zhou et al. analyzed the follow-up data of 3850 osteoarthritis patients and found that CMM treatment significantly improved the immune–inflammatory indices of these patients [208]. Moreover, Lam et al. conducted a data mining survey of 54 clinical studies and concluded that CMM is an effective adjuvant therapy for pediatric cancer patients [209]. Although there is no direct correlation between these studies and CMM, CMM represents the drugs used in TCM clinical practice, and these studies have indirectly guided the clinical application of and scientific research on CMM.

## 4. Advantages and Challenges of CADD in CMM Research

### 4.1. Advantages of CADD in CMM Research

**Figure 7:**
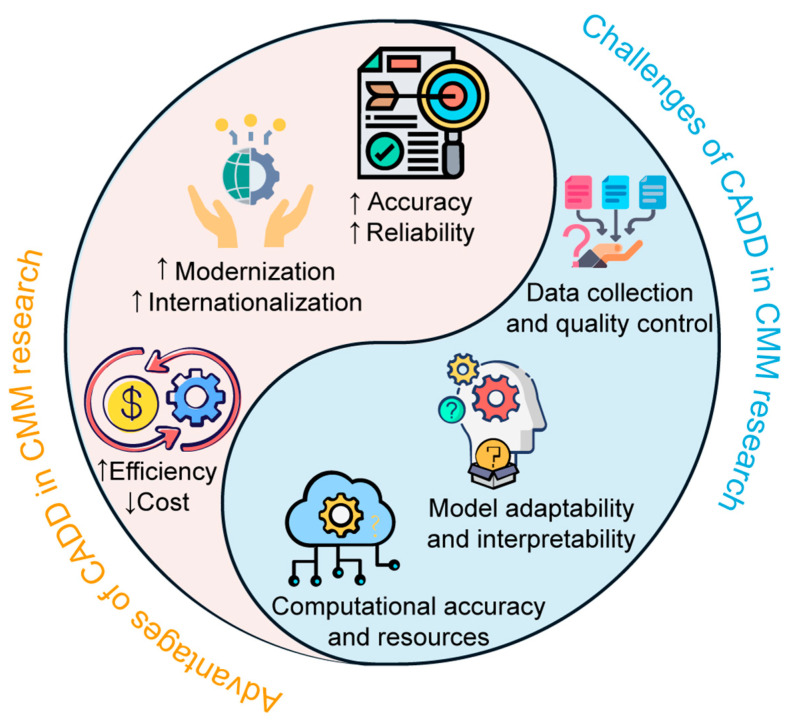
Advantages and challenges of computer-aided drug design (CADD) in Chinese materia medica (CMM) research. The “↑” means promoting effect and the “↓” means reducing effect.

The growing application of CADD in CMM research underscores the transformative potential of modern computational tools in advancing the understanding and development of ancient medical practices. This potential is evident in several key areas that highlight the advantages of CADD in CMM research (Figure 7).

#### 4.1.1. Enhancing the Accuracy and Reliability of CMM Research

Numerous case studies discussed above demonstrate that computational chemistry can yield predictive outcomes that significantly enhance the study of CAIs. The mutual validation between computational chemistry and experimental results in various scenarios strengthens the accuracy and reliability of CMM research. More importantly, the multi-ingredient, multi-target, and multi-pathway nature of CMM herbs, formulae, and Chinese patent medicines is difficult to verify through traditional experimental methods. However, informatics and AI approaches align with TCM’s holistic and systemic perspectives, offering a more robust and comprehensive understanding. Through extensive research cases, we have illustrated how these CADD techniques improve the accuracy and reliability of CMM research.

CADD also contributes to the interpretation of TCM theories, further enhancing the accuracy and reliability of CMM research. For example, TCM theory holds that CMM has properties such as four *Qi* (*Siqi* in Chinese); five flavors (*Wuwei* in Chinese); ascending, descending, floating, and sinking (*Shengjiang Fuchen* in Chinese); meridian entry (*Guijing* in Chinese); and toxicity. These properties, essential in determining the efficacy and application of CMM, have traditionally been derived from ancient texts and are challenging to explain using modern scientific principles. However, informatics and AI have successfully been employed to develop predictive models for CMM properties [210,211,212]. These models play a crucial role in bridging the gap between traditional knowledge and contemporary scientific understanding, thus guiding the direction and logic of CMM research.

#### 4.1.2. Improving the Efficiency and Reducing the Cost of CMM Research

CADD has been applied across various aspects of CMM research. While specific statistical data may be lacking, it is widely recognized that CADD effectively enhances the efficiency and reduces the costs associated with CMM research [9]. In fact, the application of CADD in WM research is more extensive and supported by more comprehensive data, which to some extent highlights the advantages of CADD in CMM research. For example, traditional drug development cycles are lengthy, typically spanning 10–15 years and costing USD 2–3 billion [213]. With the help of CADD, researchers can screen millions of molecules in just a few days and identify preclinical candidates within a year [214]. One study even estimated that AI could provide over 50 new therapies in 10 years, saving USD 50 billion in preclinical costs alone [215].

At present, traditional experimental methods still dominate CMM research, and the application of CADD is relatively limited compared to its widespread use in WM research. However, the inherent complexity of CMM, involving multiple ingredients, targets, and pathways, makes traditional experimental methods time-consuming and labor-intensive [216]. Therefore, CADD holds great promise in CMM research and is expected to evolve into a research methodology that is as significant as traditional experimental approaches.

#### 4.1.3. Promoting the Modernization and Internationalization of CMM Research

Despite the growing global acceptance of CMM, it remains regrettable that no CMM product has been approved by the FDA. Although several have entered clinical trials in recent years, with claims of potential FDA approval, they have all failed [217]. In contrast, the CMM market in China is booming, with its scale approaching CNY 1 trillion. The China Center for Drug Evaluation processed 2569 CMM registration applications in 2023, marking a year-over-year increase of 75.24% [218,219]. This discrepancy arises from the significant differences between CMM and modern chemical medicines in terms of theory, source, formulation, manufacturing, and clinical evaluation. These factors hinder the modernization and internationalization of CMM.

CADD plays a dual role in this context: on the one hand, it helps to clarify the material basis and mechanisms of CMM in treating diseases; on the other hand, it encourages CMM research to align with the processes and standards of international drug development. The data obtained from CADD in CMM research can be compared with those from WM, facilitating the modernization and internationalization of CMM. Already, CADD has helped numerous WM products to gain FDA approval. As its application in CMM research continues to expand, it is likely that, in the future, CMM products will also gain FDA approval with the assistance of CADD.

### 4.2. Challenges of CADD in CMM Research

The advantages of CADD in CMM research are very attractive; however, the practical application of CADD in CMM research has also highlighted several challenges, many of which were initially raised in WM research (Figure 7). These challenges present significant obstacles to the advancement of CADD in CMM research [10,220,221].

#### 4.2.1. Inadequate Computational Accuracy and Computational Resources

While CADD contributes to the accuracy and reliability of CMM research, particularly when experimental results are available for comparison, the general accuracy and reliability of CADD remains a subject of ongoing debate. In 1998, when Walter Kohn and John A. Pople were awarded the Nobel Prize in Chemistry for the development of DFT, the Royal Swedish Academy of Sciences stated, “chemistry is no longer a purely experimental science” [222]. Over the years, despite significant efforts by computational chemistry researchers to improve algorithms’ efficiency and accuracy, traditional experimental results continue to be regarded as the gold standard within the academic community. The most direct method of evaluating the accuracy of computational chemistry algorithms remains their consistency with experimental results. It is observed that an increasing number of researchers are adopting in silico approaches for a deeper understanding of experimental results. This has resulted in a growing body of high-quality academic literature that integrates computation with experimentation. Such a trend is expected to continue in academic research, thereby promoting the application of CADD in CMM research.

As a major country conducting CMM research, China, with its late development in the semiconductor field, heavily relies on imported computational resources, including central processing units (CPUs) and graphics processing units (GPUs). Currently, the global demand for computational resources is extremely high, fueled by the growing popularity of AI. Studies have shown that the high cost of computational resources often poses a significant challenge for researchers engaged in computational chemistry and AI [223]. In recent years, researchers have focused on minimizing computational resource usage while still achieving desired results. DeepSeek (https://www.deepseek.com/, accessed on 27 February 2025), a generative AI model developed in China, has optimized its algorithms to deliver results comparable to those of well-known generative AI models, such as ChatGPT (https://chatgpt.com/, accessed on 27 February 2025), while using significantly fewer computational resources [224].

On the other hand, the continued promise of computer-aided technologies for life sciences is evident. In 2024, the Nobel Prize in Chemistry was awarded to David Baker, Demis Hassabis, and John M. Jumper for their pioneering contributions to protein structure prediction, particularly their groundbreaking integration of AI and computational chemistry to advance protein structure research. This underscores the academic community’s confidence in the future potential of CADD. With the rapid advancement of AI, CADD has made significant strides in terms of computational accuracy. For instance, AlphaFold 3, the latest protein prediction tool, now predicts protein structures with remarkable precision [225]. Overall, although the challenge of inadequate computational accuracy and resources may complicate the widespread adoption of CADD in CMM research, it is likely that, in the near future, CADD will become as integral to CMM research as traditional experimental methods.

#### 4.2.2. Difficulties in Data Collection and Data Quality Control

The collection of data is central to informatics, which is an essential component of CADD. In terms of the database quantity, the number of CMM databases is significantly smaller than that of chemical and biological databases, and this disparity extends to other data sources, such as the literature, books, and medical cases. As a result, the application of CADD in CMM research faces inherent disadvantages in terms of data collection. CMM is characterized by its multi-ingredient, multi-target, and multi-pathway nature, often used in complex formulae in clinical practice. This complexity is reflected in the data types, which include source materials, chemical constituents, properties, efficacy, and pharmacological activity. Moreover, many of the CMM data come from ancient texts spanning various historical periods and medical schools of thought, requiring considerable human and material resources to organize and extract the information. These factors make data collection a significant challenge in CMM research.

Due to these difficulties in data collection, ensuring data quality becomes equally challenging. Our team previously compared the ingredients of the same CMM recorded in different databases and cross-referenced them with experimental data, revealing significant discrepancies [31,137]. More concerning is the fact that some TCM databases contain nearly identical data without citing specific sources, often integrated from other databases. Aside from databases, other data sources also present challenges. For example, limitations in the ancient knowledge of medicinal substances, differences in medical schools, regional and temporal variations, and constraints in recording methods have led to discrepancies between ancient records and modern scientific understanding. In some cases, records from different ancient texts even contradict one another. While it is essential for researchers to maintain high standards in the collection of primary data, we must actively employ rapidly evolving informatics and AI methods to address the difficulties in data collection and improve data quality control.

#### 4.2.3. Insufficient Adaptability and Interpretability of AI Models

Although AI has significantly improved the efficiency and accuracy of CADD, the adaptability and interpretability of AI models remain key challenges. The current AI algorithms used in CMM research heavily rely on large volumes of high-quality data for training. However, as previously discussed, data collection and quality control in CMM research are particularly challenging, which directly impacts the effectiveness and usability of model training. For instance, models often perform well only for a limited set of strictly selected ingredients and targets, making it difficult to generalize them to the complex mechanisms of CMM formulae.

AI models are often referred to as “black box” models, where researchers find it difficult to explain the biological mechanisms underlying specific prediction results [215]. In CMM-related CADD, this “black box” issue exacerbates the trust barrier that researchers face regarding a model’s predictions. Given the considerable gap between traditional CMM theory and modern scientific understanding, ensuring that a model’s output is both interpretable and verifiable in clinical applications remains a major challenge. Although some explainable AI techniques have been proposed, their application in CMM research is still in the exploratory phase and requires further development in the future [226].

## 5. Conclusions

CADD in CMM research primarily spans two key fields, computational chemistry and informatics, with the rise of AI offering new opportunities for both areas. This paper systematically reviews the foundational theories and research methods across these two domains, presenting numerous case studies directly tied to CMM research. Our analysis indicates that CADD holds significant promise in advancing CMM research, aligning well with national policies and the current trajectory of modern scientific development, particularly in the field of AI. While CADD, as a rapidly evolving technology, faces certain challenges, it is positioned to play an increasingly pivotal role in the future of CMM research.

## Figures and Tables

**Table 1:** Commonly used molecular force fields in computer-aided drug design related to Chinese materia medica research.

Force Field Name	Description	Applicable Systems	CMM Research Cases
AMBER [62]	A well-known and widely used force field for various systems. It contains many versions, such as AMBER84, AMBER86, AMBER94, AMBER96, AMBER98, AMBER99, AMBER03, AMBER03UA, AMBER99SB, AMBER99SB-ILDN, AMBER14SB, and AMBER19SB.	Proteins, nucleic acids, and some organic small molecules.	[63,64]
AMOEBA [65]	It introduces polarization effects to more accurately describe intermolecular interactions. It addresses the limitations of the traditional fixed-charge force field and proposes a more complex polarization model to improve the accuracy of the description of molecular properties.	Biological macromolecules and organic small molecules in solution environments.	[66]
CGenFF [67]	A fully CHARMM-compatible force field dedicated to the simulation of organic small molecules.	Organic small molecules.	[68,69]
CHARMM [70]	It was originally dedicated to the CHARMM program. After being updated with various versions, such as CHARMM16, CHARMM19, CHARMM22, CHARMM27, and CHARMM36, it is now supported by many programs.	Proteins, nucleic acids, phospholipids, and sugars.	[71]
GAFF [72]	It is fully compatible with the AMBER force field and can describe a variety of organic small molecules. It is a simple force field with better structural description accuracy than some complex force fields.	Organic small molecules.	[73,74]
GLYCAM [75]	It is fully compatible with the AMBER force field and can be used in the AMBER program to research glycoproteins. It includes various versions, such as GLYCAM93, GLYCAM2000, GLYCAM06, and GLYCAM06-LP.	Proteins and sugars.	[76]
GROMOS [77]	A force field with a simple energy functional and extensive applications. It contains many versions, most of which are supported only by the GROMOS program and the Gromacs program.	Condensed-phase simulation of proteins, nucleic acids, sugars, phospholipids, and organic small molecules.	[78,79]
MARTINI [80]	It improves the computational efficiency by simplifying the representation of atoms, combining multiple atoms into a single “coarse-grained” particle.	Large-scale biophysical systems such as membranes, biopolymers, and complex fluids.	[81]
MM [82]	A high-precision force field developed by the Merk Group for the simulation of organic molecules. It is suitable for conformational searches and unsuitable for condensed-phase simulations. It also includes various versions, such as MM1, MM2, MM3, MM4, MM+, and MM2X.	Organic small molecules.	[83]
MMFF94 [82]	An improved version of the MM series force fields for calculations of organic molecules and condensed phases.	Organic small molecules.	[84,85]
OPLS [86]	A force field that initially specialized in condensed-phase simulations. Its versions include OPLS-UA, OPLS-AA, and OPLS-AA/M. Starting with OPLS 2.0, the force field is exclusive to Schrödinger, Inc. and has been developed into various simulation systems.	Proteins, sugars, and organic small molecules.	[87,88]
Tripos [89]	The force field parameters are carefully optimized to provide a high-precision description based on QCC and experimental data.	Organic small molecules and proteins.	[90]

Note: Force fields unrelated to computer-aided drug design are not listed, although they may be commonly used.

## Abbreviations

The following abbreviations are used in this manuscript:

ADMET	absorption, distribution, metabolism, excretion, and toxicity
AI	artificial intelligence
AIDD	artificial intelligence drug design
CADD	computer-aided drug design
CAIs	Chinese materia medica active ingredients
CD	circular dichroism
CMM	Chinese materia medica
CMM-NP	Chinese materia medica network pharmacology
CNKI	China National Knowledge Infrastructure
CPUs	central processing units
DEGs	differentially expressed genes
DFT	density functional theory
DL	deep learning
ESP	molecular surface electrostatic potentials
FDA	US Food and Drug Administration
GO	gene ontology
GPUs	graphics processing units
GWAS	genome-wide association studies
HIV	human immunodeficiency virus
HOMO	highest occupied molecular orbital
InChI	International Chemical Identifier
IR	infrared
IRC	intrinsic reaction coordinates
KRR	kernel ridge regression
KEGG	Kyoto Encyclopedia of Genes and Genomes
LBDD	ligand-based drug design
LUMO	lowest unoccupied molecular orbital
MD	molecular dynamics
MDS	molecular dynamics simulation
ML	machine learning
MM	molecular mechanics
MM/PB(GB)SA	molecular mechanics Poisson–Boltzmann/generalized Born surface area
MS	mass spectrometry
NMR	nuclear magnetic resonance
NPA	natural population analysis
NSFC	National Natural Science Foundation of China
NPs	natural products
PPI	protein–protein interaction
QC	quantum chemistry
QCC	quantum chemical calculation
QM	quantum mechanics
QM/MM	quantum mechanics/molecular mechanics
Q-markers	quality markers
QSAR	quantitative structure–activity relationship
QSPR	quantitative structure–property relationship
SBDD	structure-based drug design
SMILES	simplified molecular input line entry system
TCM	traditional Chinese medicine
UV–Vis	ultraviolet–visible
WGCNA	weighted gene co-expression network analysis
WM	Western medicine
